# A systematic review of online resources to support patient decision-making for full-thickness rectal prolapse surgery

**DOI:** 10.1007/s10151-017-1708-7

**Published:** 2017-11-03

**Authors:** G. E. Fowler, D. M. Baker, M. J. Lee, S. R. Brown

**Affiliations:** 1 The Medical School, University of Sheffield, Beech Hill Road, Sheffield, S10 2RX UK; 2 Department of General Surgery, Sheffield Teaching Hospitals, Sheffield, UK

**Keywords:** Rectal prolapse, Surgery, Decision-making, Online resources

## Abstract

**Background:**

The internet is becoming an increasingly popular resource to support patient decision-making outside of the clinical encounter. The quality of online health information is variable and largely unregulated. The aim of this study was to assess the quality of online resources to support patient decision-making for full-thickness rectal prolapse surgery.

**Methods:**

This systematic review was registered on the PROSPERO database (CRD42017058319). Searches were performed on Google and specialist decision aid repositories using a pre-defined search strategy. Sources were analysed according to three measures: (1) their readability using the Flesch–Kincaid Reading Ease score, (2) DISCERN score and (3) International Patient Decision Aids Standards (IPDAS) minimum standards criteria score (IPDASi, v4.0).

**Results:**

Overall, 95 sources were from Google and the specialist decision aid repositories. There were 53 duplicates removed, and 18 sources did not meet the pre-defined eligibility criteria, leaving 24 sources included in the full-text analysis. The mean Flesch–Kincaid Reading Ease score was higher than recommended for patient education materials (48.8 ± 15.6, range 25.2–85.3). Overall quality of sources supporting patient decision-making for full-thickness rectal prolapse surgery was poor (median DISCERN score 1/5 ± 1.18, range 1–5). No sources met minimum decision-making standards (median IPDASi score 5/12 ± 2.01, range 1–8).

**Conclusions:**

Currently, easily accessible online health information to support patient decision-making for rectal surgery is of poor quality, difficult to read and does not support shared decision-making. It is recommended that professional bodies and medical professionals seek to develop decision aids to support decision-making for full-thickness rectal prolapse surgery.

## Introduction

Full-thickness rectal prolapse is the protrusion of the rectum beyond the anal canal. Although a benign condition, the symptoms produced by the prolapse can be debilitating and include discomfort, incontinence and constipation [1]. Although conservative management is possible, the condition can only be corrected with surgery [1]. Surgical management aims to correct the prolapse and improve functional issues. Several operations have been described for full-thickness rectal prolapse; all result in restoration of normal anatomy. These operations can be divided into two approaches, perineal and abdominal.

Abdominal approaches are typically used for physically fit patients who can tolerate a general anaesthetic. It is thought that these approaches offer good outcomes [2]. Perineal procedures can be carried out without the need for a general anaesthetic, so they are typically preferred for elderly and more unfit patients. There are concerns that this approach is associated with inferior outcomes when compared to other approaches [1–3]. More recently, improvements in anaesthetic techniques and the use of laparoscopic surgery have affected these traditional perceptions. In addition, there are multiple techniques within these groups that have various advantages and disadvantages. For instance, perineal procedures may involve rectal amputation with a potential increased risk of complications, or preservation of the rectum with a higher risk of functional issues. Abdominal procedures may involve a mesh support with the theoretical reduction in incontinence but increased risk of harm. There is also equipoise amongst surgeons as to the optimum approach. The surgical management of full-thickness rectal prolapse is therefore complex, and these considerations need to all be carefully considered with the patient in the shared decision-making process.

Given the relevant trade-offs for each approach and procedure, it is important to take patient preferences into account when planning surgery. The consent paradigm has shifted to encourage a shared-decision model [4]. Surgeons are encouraged to allow time for patients to read further material on their condition and treatment, including accessing online information [4]. It has been shown that around two-thirds of patients go online to seek health information [5, 6]. It is therefore essential that this information is of high quality, as it can significantly support and improve patients’ experiences with decision-making [7]. It has also been documented that the quality of online health information to aid patient decision-making is variable [7–12].

The aim of this study was to assess the readability and quality of online resources to support patient decision-making for full-thickness rectal prolapse surgery.

## Materials and methods

This project was registered (ref CRD42017058319) on the international database PROSPERO (https://www.crd.york.ac.uk/PROSPERO/). The review was reported in concordance with the Preferred Reporting Items for Systematic Reviews and Meta-Analyses (PRISMA) guidance [13]. The methods in this study are adapted from two recent studies assessing the quality of online health information to support patient decision-making in colorectal surgery [9, 10].

### Search strategy

Searches were performed on Google (www.google.co.uk; Mountain View, CA, USA) and specialist decision aid repositories, which included the Decision Aids Library Inventory (DALI), NHS Choices, NHS Evidence, Clinical Trials Gateway and the National Guidance Clearing House.

All searches used the following four pre-defined search strings: ‘rectal prolapse’, ‘rectal prolapse surgery’, ‘rectal prolapse operation’ and ‘rectal prolapse repair’. These lay searches aimed to capture a typical patient search, an approach adapted from similar studies in colorectal disease [10, 12], and expanded from a previous rectal prolapse study [11]. Searches from the repositories included any online decision aids. Searches performed on Google included all sources found on the first two pages, since 92% of online users do not go beyond the first page [14]. No other search engines were used, as Google is the most popular search engine for accessing online health information [14]. Google searches were done using the ‘Incognito window’ to avoid the effects of ‘Google personalisation’, which provides the most relevant results based on your search history [15].

### Data extraction

Data extraction was performed by two authors (GEF and DMB), and any conflicts were resolved by a third author (MJL). Data were extracted onto a Microsoft Excel 2013 (Microsoft, Washington, USA) spreadsheet and included information on the sources URL, upload source, country of origin and descriptions on the management of a full-thickness rectal prolapse, both medical and surgical.

### Eligibility criteria

The extracted data were screened for duplicates, and these sources were subsequently removed. Sources included for full-text analysis were assessed against the following pre-defined inclusion criteria;

1. Sources with the content about full-thickness rectal prolapse and surgery.
2. Content in English language.
3. Content aimed at patients.

Sources were excluded if they did not meet the inclusion criteria or met any of the following pre-defined exclusion criteria: advertisements, online videos (e.g. YouTube videos), newspaper articles, academic sources not aimed at patients (e.g. PubMed articles) and sources requiring a subscription.

### Data analysis

Sources were analysed according to three measures;

1. The readability using the Flesch–Kincaid Reading Ease score (https://www.webpagefx.com/tools/read-able/). This measures the reading ease, with a score from 0 to 100 corresponding to the education level required to read the information based on the US schools grading system. A low score suggests the text is complicated to understand, and a score between 60 and 80 suggests the text is easy to read by a 12- to 15-year-old [16].
2. DISCERN score. The DISCERN instrument is designed to assess the quality of written information on the treatment choices for a defined health problem [17]. The tool consists of 15 questions, each rated on a 5-point scale of 1 (quality criterion not fulfilled), 2–4 (quality criterion partially fulfilled) and 5 (quality criterion completely fulfilled). A global score is also given and is rated on a 3-point scale of 1 (serious shortcomings), 3 (potentially important shortcomings) or 5 (minimal shortcomings). The score indicates the assessor’s overall conclusion about the quality of the source for providing information on the treatment choices for the defined health problem.
3. International Patient Decision Aids Standards (IPDAS) minimum standards criteria score (IPDASi, v4.0). The IPDASi criteria consist of three categories: *qualifying*, *certifying* and *quality* criteria [18]. These categories aid the assessment of patient decision aids. The last category is considered desirable, but not essential for the assessment of patient decision aids, and was therefore excluded from this study, leaving a 12-item checklist of qualifying and certifying criteria [18].

## Results

### Website selection

**Fig. 1:**
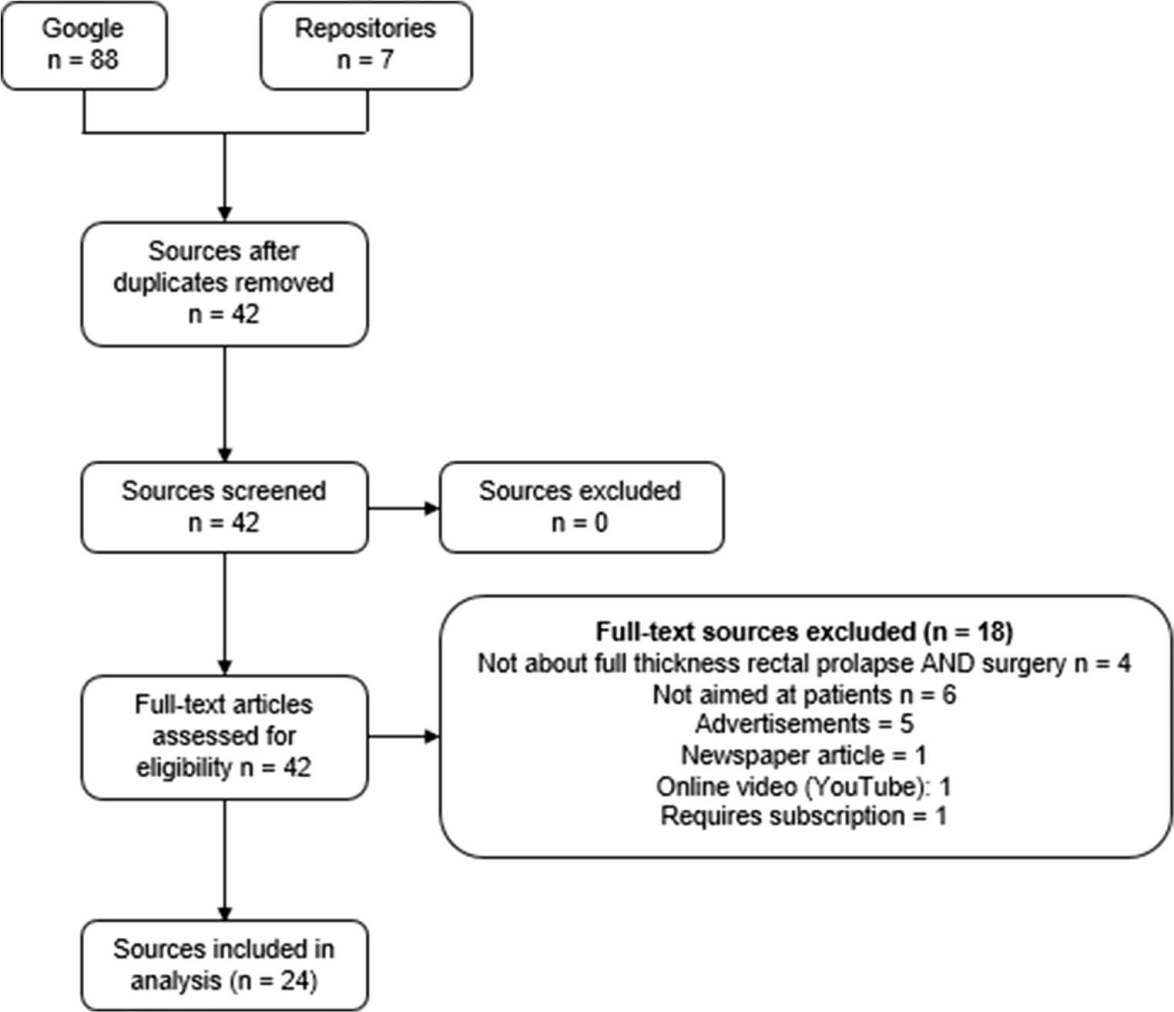
PRISMA flow diagram of search strategy

The lay search strings found a total of 3,497,000 websites from Google and 7 from the specialist decision aid repositories (Fig. 1). There were 88 sources found from the first two pages of Google, with 53 duplicates removed from Google, or the repositories. This left 42 sources for full-text assessment, of which 18 did not meet the pre-defined eligibility criteria. This left 24 sources for inclusion in the review, all of which were identified through Google. Sources included in the study, and procedures discussed, are presented in Table 1.

**Table 1:** Summary of surgical procedures described

Source and URL Link	Abdominal procedures described for full-thickness rectal prolapse	Perineal procedures described for full-thickness rectal prolapse
(1) patient.info/doctor/rectal-prolapse	Anterior resection, Marlex rectopexy, suture rectopexy and resection rectopexy, laparoscopic surgical rectopexy, combined laparoscopic–perineal procedure	Altemeier procedure, Délorme’s procedure, Thiersch procedure
(2) http://www.bowelcancerresearch.org/about-bowels/bowel-conditions/rectal-prolapse/	Abdominal surgery was only mentioned (and in combination with perineal surgery)	Perineal surgery was only mentioned
(3) http://www.bupa.co.uk/health-information/directory/r/rectal-prolapse	Abdominal surgery was only mentioned	Perineal surgery was only mentioned
(4) www.emedicinehealth.com/rectal_prolapse/page2_em.htm	Abdominal surgery was only mentioned	Perineal surgery was only mentioned
(5) www.webmd.com/digestive-disorders/tc/rectal-prolapse-topic-overview#1	Anterior resection, Marlex rectopexy	None
(6)emedicine.medscape.com/article/2026460-treatment	Anterior resection, Marlex rectopexy, suture rectopexy, resection rectopexy, Laparoscopic surgical rectopexy,	Altemeier procedure, Délorme’s procedure, Thiersch procedure
(7) en.wikipedia.org/wiki/Rectal_prolapse	Rectopexy, laparoscopically	Altemeier procedure, Délorme’s procedure, Thiersch procedure
(8) www.mayoclinic.org/tests-procedures/rectal-prolapse-surgery/details/what-you-can-expect/rec-20259132	Marlex rectopexy, laparoscopically	Altemeier procedure, Délorme’s procedure
(9) http://www.birminghambowelclinic.co.uk/rectal-prolapse/	Rectopexy, ventral mesh rectopexy, laparoscopically	Altemeier procedure, Délorme’s procedure
(10) http://www.surgwiki.com/wiki/Rectal_prolapse	Sacral rectopexy, Frykman-Goldberg operation, mesh rectopexy	Altemeier procedure, Délorme’s procedure
(11) my.clevelandclinic.org/health/articles/rectal-prolapse	Rectopexy, resection	Altemeier procedure, Délorme’s procedure
(12) http://www.bmihealthcare.co.uk/treatments/colorectal-surgery/surgery-for-rectal-prolapse	Abdominal surgery was only mentioned (open/laparoscopic)	Perineal procedure mentioned only
(13) http://www.mayoclinic.org/tests-procedures/rectal-prolapse-surgery/home/ovc-20259104	Rectopexy, laparoscopically	Altemeier procedure, Délorme’s procedure
(14) medlineplus.gov/ency/article/002932.htm	Rectopexy	Altemeier procedure
(15) http://sussexsurgical.co.uk/information.php?t=Prolapse&s=Bowel-Conditions&id=62	Sutured and mesh rectopexy fixation, laparoscopically	Altemeier procedure, Délorme’s procedure
(16) http://www.royalberkshire.nhs.uk/patient-information-leaflets/Surgery%20info_delorme-surgery-for-rectal-prolapse-september-2014.htm	None	Délorme’s procedure
(17) http://www.surgeryencyclopedia.com/Pa-St/Rectal-Prolapse-Repair.html	Rectopexy, anterior resection, laparoscopically	Altemeier procedure, Délorme’s procedure, Thiersch procedure
(18) http://www.betterhealth.vic.gov.au/health/conditionsandtreatments/rectal-prolapse	Rectopexy, anterior resection, laparoscopically	Altemeier procedure
(19) colorectalsurgeonssydney.com.au/?page_id=432	Ventral mesh rectopexy,, laparoscopically, robotic ventral mesh rectopexy	Altemeier procedure, Délorme’s procedure
(20) http://www.nuffieldhealth.com/treatments/rectal-prolapse-repair	Rectopexy, laparoscopic rectopexy	Altemeier procedure, Délorme’s procedure
(21) http://www.birminghambowelclinic.co.uk/treatments/delormes-procedure/	None	Délorme’s procedure
(22) www.bladderandbowelfoundation.org/bowel/bowel-treatments/rectal-prolapse-repair/	Rectopexy	Délorme’s procedure
(23) www.royalberkshire.nhs.uk/patient-information-leaflets/Surgery_Laparascopic%20keyhole%20rectopexy%20rectal%20repair%20surgery%20sept%202014.htm	Laparoscopic rectopexy	None
(24) http://www.fascrs.org/patients/disease-condition/rectal-prolapse-expanded-version	Rectopexy	Altemeier procedure, Délorme’s procedure

### Website characteristics

The website characteristics are summarised in Table 2. The majority of websites were based in the UK (*n* = 11), with the remainder from the USA (*n* = 8), Australia (*n* = 3) or undetermined (*n* = 2). The uploaded sources were healthcare service providers (*n* = 12), patient resources (*n* = 5), public domains (*n* = 2), charities (*n* = 2), governmental or professional organisations (*n* = 2) or other (*n* = 1). Thirteen of these sources described both the medical and surgical treatments, and 11 described only the surgical treatment. Four of the latter stated explicitly the consequences for a patient deciding not to have surgery.

**Table 2:** Summary of website characteristics and Flesch–Kincaid Reading Ease scores

Source and URL Link	Upload source	Country of origin	Described treatment (medical/surgical/both)	Flesch–Kincaid Reading Ease Scores
(1) patient.info/doctor/rectal-prolapse	Patient	UK	Both	42.6
(2) http://www.bowelcancerresearch.org/about-bowels/bowel-conditions/rectal-prolapse/	Bowel and Cancer Research	UK	Both	66.8
(3) http://www.bupa.co.uk/health-information/directory/r/rectal-prolapse	Bupa	UK	Both	61.4
(4) www.emedicinehealth.com/rectal_prolapse/page2_em.htm	eMedicineHealth	USA	Both	52.8
(5) www.webmd.com/digestive-disorders/tc/rectal-prolapse-topic-overview#1	WebMD	USA	Both	62.3
(6) emedicine.medscape.com/article/2026460-treatment	Medscape	USA	Both	29.7
(7)en.wikipedia.org/wiki/Rectal_prolapse	Wikipedia	N/A	Both	39.7
(8) www.mayoclinic.org/tests-procedures/rectal-prolapse-surgery/details/what-you-can-expect/rec-20259132	Mayo Clinic	USA	Surgical	48.5
(9) http://www.birminghambowelclinic.co.uk/rectal-prolapse/	Birmingham Bowel Clinic	UK	Surgical	31.5
(10) http://www.surgwiki.com/wiki/Rectal_prolapse	SurgWiki	AUS	Both	29.2
(11) my.clevelandclinic.org/health/articles/rectal-prolapse	Cleveland Clinic	USA	Both	48.7
(12) http://www.bmihealthcare.co.uk/treatments/colorectal-surgery/surgery-for-rectal-prolapse	BMI Healthcare	UK	Surgical	55.8
(13) http://www.mayoclinic.org/tests-procedures/rectal-prolapse-surgery/home/ovc-20259104	Mayo Clinic	USA	Surgical	47.7
(14) medlineplus.gov/ency/article/002932.htm	Medline Plus	USA	Surgical	56.8
(15) http://sussexsurgical.co.uk/information.php?t=Prolapse&s=Bowel-Conditions&id=62	Sussex Surgical	UK	Surgical	34.5
(16) http://www.royalberkshire.nhs.uk/patient-information-leaflets/Surgery%20info_delorme-surgery-for-rectal-prolapse-september-2014.htm	Royal Berkshire NHS Foundation Trust	UK	Surgical	70.7
(17) http://www.surgeryencyclopedia.com/Pa-St/Rectal-Prolapse-Repair.html	Encyclopedia of Surgery	–	Both	70.9
(18) http://www.betterhealth.vic.gov.au/health/conditionsandtreatments/rectal-prolapse	Better Health Channel	AUS	Both	25.2
(19) colorectalsurgeonssydney.com.au/?page_id=432	Colorectal Surgeons Sydney	AUS	Both	39.0
(20) http://www.nuffieldhealth.com/treatments/rectal-prolapse-repair	Nuffield Health	UK	Both	56.8
(21) http://www.birminghambowelclinic.co.uk/treatments/delormes-procedure/	Birmingham Bowel Clinic	UK	Surgical	39.2
(22) www.bladderandbowelfoundation.org/bowel/bowel-treatments/rectal-prolapse-repair/	Bladder & Bowel Community	UK	Surgical	30.7
(23) www.royalberkshire.nhs.uk/patient-information-leaflets/Surgery_Laparascopic%20keyhole%20rectopexy%20rectal%20repair%20surgery%20sept%202014.htm	Royal Berkshire Hospital (NHS Foundation Trust)	UK	Surgical	85.3
(24) http://www.fascrs.org/patients/disease-condition/rectal-prolapse-expanded-version	American Society of Colon and Rectal Surgeons	USA	Surgical	45.5

### Readability

The mean Flesch–Kincaid Reading Ease score was 48.8 (SD ± 15.6, range 25.2–85.3) out of 100, representing a reading level expected at university [16]. This is higher than recommended for patient education materials, which recommends a score between 80 and 100 [19].

### DISCERN score

The DISCERN scores are presented on a visual analogue scale in Table 3. Scores of 1, 3 and 5, respectively, indicate whether the source met none, partially met or all of the criteria to the question. The overall quality of online resources on treatment choices for full-thickness rectal prolapse were poor in this study, with a median DISCERN score of 1/5 (SD ± 1.18, range 1–5) out of 5. Only one source scored highly on its overall quality (5/5). This was uploaded by the American Society of Colon and Rectal Surgeons (source 24). All other sources scored poorly in their overall quality, meeting none of the criteria in several of the domains. Only 11/24 sources partially met all of the criteria (overall quality score of 3/5), and with the exception of one source (16), none of eleven sources provided clear aims or achieved their aims. This highlights serious or potentially important shortcomings on the written information on treatment choices for full-thickness rectal prolapse.

**Table 3:**
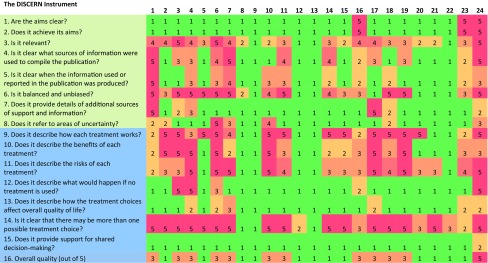
DISCERN Scores

The colour of the square visually represents the DISCERN score for each domain for a particular source: red = 5, dark orange = 4, orange = 3, pale orange = 2, green = 1

Generally, the sources scored poorly on questions important for shared decision-making (questions 12, 13 and 15), including on how the treatment choice will affect overall quality of life and the consequences of not choosing a treatment. On the latter, only three sources scored highly (5/5, sources 3–4 and 24). Domains which scored more highly were questions on how the treatment works, the benefits and risks of each and for providing more than one treatment choice (questions 9, 10, 11 and 14). There were two sources (9 and 14) which scored poorly (1/5) in all domains. They were surgically focused private healthcare resources and provided no descriptions on the medical management of full-thickness rectal prolapse.

### IPDASi (v4.0) minimum standards criteria

The IPDASi scores are shown in Table 4. A check mark indicates whether the criteria have been met, producing a total score out of 12. All criteria should be met to be in concordance with IPDAS recommendations to meet minimum decision-making standards [18]. The median IPDASi score was 5/12 (SD ± 2.01, range 1–8), with none of the sources meeting all of the criteria, or describing the experience of the consequence of treatment options (criteria 6), or providing a publication date (criteria 9). Domains which scored more highly included descriptions of the health condition (21/24 sources), the treatment options (22/24 sources), with pros and cons (16/24 and 17/24, respectively), while sources which scored the poorest in all of the IPDASi minimum standards criteria (1/12) were surgically focused (12, 21–22). There were only five sources (1, 6–7, 13, 24) which provided citation to their evidence, and this included the four sources (1, 6–7, 24) which had the highest IPDASi minimum standards criteria scores, of which three of the four (1, 6–7) described both the medical and surgical treatments for full-thickness rectal prolapse.

**Table 4:** IPDAS minimum standards criteria

Qualifying Criteria	1	2	3	4	5	6	7	8	9	10	11	12	13	14	15	16	17	18	19	20	21	22	23	24
1. Describes health condition	✓	✓	✓	✓	✓	X	✓	✓	✓	✓	✓	✓	✓	✓	✓	✓	✓	✓	✓	✓	X	X	✓	✓
2. States explicit decision	X	X	✓	X	X	X	X	X	X	X	X	X	X	X	X	X	X	X	X	X	X	X	✓	✓
3. Describes options	✓	✓	✓	✓	✓	✓	✓	✓	✓	✓	✓	X	✓	✓	✓	✓	✓	✓	✓	✓	X	✓	✓	✓
4. Describes positive features	✓	✓	✓	✓	X	✓	✓	X	X	✓	✓	X	✓	✓	✓	✓	✓	✓	✓	X	X	X	X	✓
5. Describes negative features	✓	✓	✓	✓	X	✓	✓	X	X	✓	✓	X	✓	✓	X	✓	X	✓	✓	✓	✓	X	✓	✓
6. Describes the experience of the consequence of options	X	X	X	X	X	X	X	X	X	X	X	X	X	X	X	X	X	X	X	X	X	X	X	X
Certifying Criteria																								
7. Balanced and equal detail for all options	✓	✓	✓	✓	✓	✓	✓	X	X	✓	✓	X	✓	✓	✓	X	✓	✓	✓	✓	X	X	X	✓
8. Citation to evidence	✓	X	X	X	X	✓	✓	X	X	X	X	X	✓	X	X	X	X	X	X	X	X	X	X	✓
9. Publication date provided	X	X	X	X	X	X	X	X	X	X	X	X	X	X	X	X	X	X	X	X	X	X	X	X
10. Update policy provided	✓	X	X	X	X	✓	X	✓	X	✓	✓	X	X	✓	X	✓	X	✓	X	X	X	X	X	X
11. Information about levels of uncertainty around event	X	X	X	X	X	✓	✓	X	X	X	X	X	X	X	X	X	X	X	X	X	X	X	X	X
12. Funding source	✓	X	X	X	X	X	X	X	X	X	X	X	X	X	X	X	X	X	X	X	X	X	X	X
Total/12	8	5	6	5	3	7	7	3	2	6	6	1	6	6	4	5	4	6	5	4	1	1	4	7

## Discussion

This study has systematically assessed the readability and quality of online health resources to support patient decision-making for full-thickness rectal prolapse surgery. Of these sources, the average readability was higher than recommended for patient education materials, and none met minimum decision-making standards.

The overall poor quality is consistent with findings presented by Sehgal et al. [11], which reported the poor quality of online patient information for full-thickness rectal prolapse, but based results on the DISCERN instrument alone. The data from our study are of additional value, as it also provides the additional dimensions of Flesch–Kincaid Reading Ease and IPDASi scores.

Poor quality of online health information about other conditions including breast cancer [7], perianal Crohn’s fistula [9] and ulcerative colitis [10] has been reported. This is concerning, as patients may trust in these sources of information. A European survey found 90% of patients were satisfied with online health information, with only a small percentage (10%) not satisfied on the grounds of reliability, commercially orientated or not enough detail [6].

In our study, private healthcare resources describing only the surgical management of full-thickness rectal prolapse had the poorest quality of written information, indicated by low DISCERN and IPDAS scores and high Flesch–Kincaid Reading Ease scores. In addition, these sources did not support shared decision-making, as seen in previous work [7, 9–11]. Only one source evaluated by the DISCERN instrument was of high overall quality, but remained difficult to read. The source was provided by the American Society of Colon and Rectal Surgeons. Although this may seem an obvious source of reliable information by healthcare professionals, this may be less obvious to patients.

An online decision aid to support patient decision-making for full-rectal prolapse surgery although perhaps desirable is nevertheless difficult to create for rectal prolapse since the management may be complex, and many of the surgical decisions are based on surgical dogma rather than evidence base [1]. The consultation is therefore still the most powerful tool to support patient decision-making for full-thickness rectal prolapse, as the online health information is currently of poor quality and not commonly read by the patients likely to present with this condition.

This study has some limitations. Online videos were not analysed despite their increasing popularity as a source of patient information [20]. Their popularity for the patients likely to present with a full-thickness rectal prolapse is not yet reported and is presumably even less commonly used than written online health information. We only used the search engine Google as there is evidence that Google incorporates the vast majority of websites found from other search engines [21]. Restricting the searches to only the first two pages of Google was another limitation. However, most online users do not go beyond the first page [14]. There are also limitations from using the DISCERN instrument. It may overestimate the overall quality of online health information to support patient decision-making [9, 10]. Sources scored highly if they mentioned more than one possible treatment option, but can also score highly if they mention only one other treatment option, without reference to medical alternatives, or explaining the pros and cons of the other treatment option. A particular issue with rectal prolapse and online health information is the age distribution of the condition. Most patients are elderly with a peak incidence in the seventh decade [1]. Only a small portion of patients above the age of 55 use online health information [6].

Conversely, our study has several strengths. To our knowledge, it is the first systematic review of online resources to support patient decision-making for full-thickness rectal prolapse surgery to have followed PRISMA guidance and provided Flesch–Kincaid Reading Ease and IPDASi scores.

Clearly, there is work to be done to improve patient information. Professional organisations should engage with patients to understand their information needs and develop an appropriate resource to support this. This could take the form of a simple leaflet, an option matrix or an online decision support tool. Other work to explore patient values and ‘trade-offs’ would make this of more use to support patients. In the meantime, clinicians should identify a reliable online resource for the condition and guide patients towards this.

## Conclusions

Easily accessible online health information to support patient decision-making for rectal surgery is of poor quality, difficult to read and does not support shared decision-making. It is recommended that professional bodies and medical professionals seek to develop decision aids to support decision-making for full-thickness rectal prolapse surgery and in other areas of medicine.
